# Impact of Cyberprogram 2.0 on Different Types of School Violence and Aggressiveness

**DOI:** 10.3389/fpsyg.2016.00428

**Published:** 2016-03-30

**Authors:** Maite Garaigordobil, Vanesa Martínez-Valderrey

**Affiliations:** Personality, Assessment and Psychological Treatments, Faculty of Psychology, University of the Basque CountrySan Sebastián, Spain

**Keywords:** bullying, cyberbullying, school violence, adolescence, aggressiveness

## Abstract

Some antibullying interventions have shown positive outcomes with regard to reducing violence. The aim of the study was to experimentally assess the effects on school violence and aggressiveness of a program to prevent and reduce cyberbullying. The sample was comprised of a randomly selected sample of 176 adolescents (93 experimental, 83 control), aged 13–15 years. The study used a repeated measures pre-posttest design with a control group. Before and after the program, two assessment instruments were administered: the “Cuestionario de Violencia Escolar-Revisado” (CUVE-R [School Violence Questionnaire – Revised]; Álvarez-García et al., 2011) and the “Cuestionario de agresividad premeditada e impulsiva” (CAPI-A [Premeditated and Impulsive Aggressiveness Questionnaire]; Andreu, 2010). The intervention consisted of 19 one-hour sessions carried out during the school term. The program contains 25 activities with the following objectives: (1) to identify and conceptualize bullying/cyberbullying; (2) to analyze the consequences of bullying/cyberbullying, promoting participants’ capacity to report such actions when they are discovered; (3) to develop coping strategies to prevent and reduce bullying/cyberbullying; and (4) to achieve other transversal goals, such as developing positive variables (empathy, active listening, social skills, constructive conflict resolution, etc.). The pre-posttest ANCOVAs confirmed that the program stimulated a decrease in: (1) diverse types of school violence—teachers’ violence toward students (ridiculing or publicly humiliating students in front of the class, etc.); students’ physical violence (fights, blows, shoves… aimed at the victim, or at his or her property, etc.); students’ verbal violence (using offensive language, cruel, embarrassing, or insulting words… toward classmates and teachers); social exclusion (rejection or exclusion of a person or group, etc.), and violence through Information and Communication Technologies (ICT; violent behaviors by means of electronic instruments such as mobile phones and the Internet)—; and (2) premeditated and impulsive aggressiveness. Pre-posttest MANCOVA revealed differences between conditions with a medium effect size. This work contributes an efficacious intervention tool for the prevention and reduction of peer violence. The conclusions drawn from this study have interesting implications for educational and clinical intervention.

## Introduction

The prevalence of school bullying is verified worldwide, with no notable differences due to geographical, cultural, or educational context. Aside from the debate about the possible importance of numbers, actually, in all schools, some people are suffering their peers’ bullying, and other people are acquiring antisocial behaviors. The highly negative consequences for all of the victims and aggressors involved oftentimes endure for the rest of their lives. The problems that arise of bullying and cyberbullying (anxiety, depression, stress, somatization, academic problems, suicide, violence, …) entail long-lasting and extensive effects that must be faced.

Reviews of studies on the consequences of bullying and cyberbullying (Garaigordobil, 2011) have shown that peer violence has extremely negative consequences for all involved, although with diverse symptoms and levels of suffering. The most marked effects are observed in the victims, but the aggressors and observers are also the recipients of learning experiences and negative habits. Regardless of their role, all those involved in abusive situations are at greater risk of suffering from psychosocial maladjustment and psychopathological disorders in adolescence and adulthood. No doubt, the most extreme consequence of peer bullying (bullying/cyberbullying) is the victim’s suicide or death, and this was precisely what prompted the first investigation carried out in Norway by Olweus (1973).

Victims of cyberbullying suffer the same or even greater psychological harm than victims of face-to-face bullying: the damaging information is available to everyone 24 h a day, the bullies are often anonymous, the victimization process is continuous and unavoidable, and most of the time, it is very difficult to eliminate the published material, which is usually publicly accessible for long periods of time. Adolescents tend to be reluctant to talk to adults about the abuse they are suffering due to the emotional trauma, their fear that adults will think they are to blame, fear of possible “revenge,” or concern that their use of the Internet or mobile phone will be restricted (Garaigordobil, 2011).

There are many studies showing that the victims have depressive symptoms (Erdur-Baker and Tanrikulu, 2010; Estévez et al., 2010; Hinduja and Patchin, 2010). Kowalski et al. (2008) reviewed the literature about the effects of cyberbullying for the victims, concluding that these effects may be similar to those identified in traditional bullying (depression, low self-esteem, helplessness, social anxiety, poor concentration, poor academic performance, suicidal ideas, etc.). In this direction, in the study of Sourander et al. (2010), being a victim was associated with emotional problems, headaches, recurrent stomach aches, sleeping problems, and a feeling of insecurity at school. Being a bully was associated with hyperactivity, behavioral problems, low prosocial behavior, and regular alcohol and tobacco abuse. At its most extreme point, cyberbullying can lead to suicide and youth violence (Feinberg and Robey, 2009). Therefore, measures of educational styles and of awareness of the phenomena, involving schools, students, and their families, are needed (Garaigordobil, 2011; Ortega et al., 2012; Pronk et al., 2013).

This study was motivated by the concern for peer violence expressed by parents, teachers, and society in general. Other forms of bullying are currently emerging, such as cyberbullying, which consists of using ICT, mainly the Internet (e-mail, SMS, websites, blogs…) and mobile phones, to perpetrate peer bullying. Cyberbullying consists of aggressive and intentional behavior repeated over time against a victim who cannot easily defend him- or herself. Various means are used for bullying: SMS, telephone bullying (anonymous calls…), recordings of acts of physical aggression or humiliation disseminated by mobile phone or the Internet, bullying by means of photographs and videos distributed by mobile phone or uploaded to YouTube, e-mails, instant messages, social networks, websites, etc. (Smith et al., 2008).

Reviews of studies analyzing the prevalence of cyberbullying showed that violence through ICT has recently become a relevant problem in all developed countries. The rapid development and growth of this new form of harassment has generated the urgent need for prevention and intervention. Empirical evidence suggests the need for active prevention and intervention strategies in educational, family, and clinical-therapeutic settings.

Some antibullying interventions have shown positive outcomes in reducing violence (Sapouna et al., 2010). In recent years, efficacious programs were developed to prevent and reduce bullying, which have improved the social climate of the classroom and the school (Olweus, 1991; Olweus and Limber, 2010a,b; Rawana et al., 2011), intragroup relations (Fekkes et al., 2006), and the feeling of safety at school (Heydenberk et al., 2006).

In addition, diverse antibullying programs have decreased aggressive behavior outside of the classroom, disruptive behaviors in the classroom (Fonagy et al., 2005; Twemlow et al., 2005), the perception of aggressiveness in class, fights (Heydenberk et al., 2006), aggressiveness (Grossman et al., 1997; McMahon et al., 2000; Ortega and Lera, 2000; Orpinas et al., 2003), reinforcement of aggressive behavior (Olweus, 2004; Kärnä et al., 2009; Williford et al., 2012), antisocial and violent behavior (Olweus, 1991; Menard et al., 2008; Olweus and Limber, 2010a,b), and bullying (assisting and reinforcing the bully; Kärnä et al., 2013). Therefore, there is some evidence that antibullying programs decrease aggressiveness.

Also, some programs to prevent and reduce cyberbullying that have been experimentally assessed have shown positive effects. The *Brief Internet Cyberbullying Prevention Program* (Doane, 2011), carried out with 375 Canadian students, had three axes: (a) real news items about cyberbullying victims; (b) definition, types, situations, and prevalence of cyberbullying; and (c) cases of cyberbullying from the victims’ viewpoint. The results showed that the intervention decreased the behaviors of perpetration of cyberbullying and positive attitudes toward this type of behavior, while knowledge about cyberbullying increased.

*The WebQuest Cyberbullying Prevention Course* (Lee et al., 2013) was carried out with 61 Taiwanese seventh-grade students. The experimental group received eight sessions of the teaching intervention. WebQuest is a set of student-centered and exploration-oriented learning activities presented through a webpage layout. Based mainly on social constructivism and collaborative learning theory, WebQuest has six components: introduction, task, process, resources, assessments, and conclusions. The results showed that the WebQuest course enhanced knowledge of cyberbullying, reduced intentions, and retained the effects after the learning.

*The KiVa Antibullying Program* (Williford et al., 2013) intervention program for children and adolescents has four levels: school, classroom, individual, and teachers. The comprehensive approach contains diverse strategies: support to victims and aggressors, manuals, website discussion forum for teachers, information for parents, increased supervision, and creation of virtual, Internet-based environments. The study examined differences in the frequencies of cyberbullying and cybervictimization between intervention (*n* = 9,914) and control (*n* = 8,498) students. The participants were fourth- to ninth-grade students in Finland. Results revealed a significant intervention effect on the frequency of cybervictimization (KiVa students reported lower frequencies of cybervictimization at posttest than students in a control condition).

The *Media Heroes Cyberbullying Prevention Program* (Chaux et al., 2016) promotes empathy, knowledge of risks and consequences, and strategies that allow bystanders to defend victims from cyberbullying. The study was carried out with 722 German students (ages 11–17), applying 15 intervention sessions. The results confirmed that participating in Media Heroes led to a reduction in traditional bullying perpetration (but not in victimization), in addition to previously reported reduction of cyberbullying perpetration.

However, it is important to note that the results of antibullying programs are inconsistent. Some show evidence of positive effects (Williford et al., 2012; Kärnä et al., 2013) but others, contrary to expectations, show increases in bullying (see review of Jeong and Lee, 2013). Even Olweus’ program has not been replicated successfully. In this sense, some meta-analyses disagree with these programs’ effectiveness. Ferguson et al. (2007) concluded that global antibullying programs generate little discernible effect on young participants, whereas Ttofi and Farrington (2011) showed that global school-based antibullying programs are effective.

Although many studies suggest that various types of peer victimization among schoolchildren are declining (Finkelhor et al., 2010), bullying is still a problem in schools. The rapid growth of cyberbullying, this new form of bullying, its high prevalence worldwide and extremely negative consequences on all those involved has generated the urgent need to propose programs to prevent and/or intervene in this type of violence (Garaigordobil, 2011, 2013). In spite of its growing social relevance and the variety of existing resources and protocols, literature reviews show that there are currently very few validated psychoeducational intervention programs aimed at preventing, reducing, intervening in, and palliating the effects of cyberbullying.

Therefore, the aim of the study was to experimentally assess the effects of a program (Cyberprogram 2.0; Garaigordobil and Martínez-Valderrey, 2014a) to prevent and reduce cyberbullying on school violence (teachers’ violence toward students, students’ physical and verbal violence, social exclusion, disruption in the classroom, violence by means of ICT) and aggressiveness. This study is part of a broader investigation that implemented the Cyberprogram 2.0 and assessed its effects on many dependent variables. The study presented herein complements previous evaluations that confirmed that the program stimulated a significant increase of positive social behaviors (social conformity, help-collaboration, self-assurance-firmness, prosocial leadership) (Garaigordobil and Martínez-Valderrey, 2014b), decrease of the amount of bullying and cyberbullying behaviors received and/or perpetrated, increase of the capacity for empathy (Garaigordobil and Martínez-Valderrey, 2015a), increase of cooperative conflict-resolution strategies, decrease of aggressive and avoidant strategies, and increase of self-esteem (Garaigordobil and Martínez-Valderrey, 2015b).

The investigation applies a cognitive-behavioral theoretical framework to analyze the effect of the intervention (Cyberprogram 2.0). The program promotes cognitive restructuring of the roles involved in bullying/cyberbullying situations. Specifically, it stimulates the modification of cognitions, which in turn fosters changes at the behavioral level; that is, victims learn to defend themselves and observers intervene in favor of the victims.

Some anti-cyberbullying programs (Doane, 2011; Lee et al., 2013; Williford et al., 2013) have promoted a reduction of technological bullying, antibullying programs have stimulated decreases of face-to-face bullying (Kärnä et al., 2013), of aggressive behavior in general (e.g., McMahon et al., 2000; Ortega and Lera, 2000; Orpinas et al., 2003; Fonagy et al., 2005), and other socio-emotional intervention programs (Garaigordobil et al., 2009; Garaigordobil and Peña-Sarrionaindia, 2015) and previous assessments of Cyberprogram 2.0 (Garaigordobil and Martínez-Valderrey, 2014b, 2015a,b) found no gender differences in the effects of the intervention. Therefore, this study proposes three hypotheses as regards the intervention: (H1) It will decrease diverse types of school violence (teachers’ violence toward students, students’ physical and verbal violence, social exclusion, disruption in the classroom, violence through ICT or cyberbullying); (H2) It will decrease impulsive and premeditated aggressiveness; and (H3) It will affect both sexes similarly.

## Materials and Methods

### Participants

This study sample included 176 adolescents, aged between 13 and 15 years, who studied Secondary Education (grade 8); 77 (43.8%) males and 99 (56.3%) females. Of the initial 178 adolescents, two moved to another school before completing the program. Of the total sample, 93 (52.8%) were randomly assigned to the experimental condition, and 83 (47.2%) to the control condition. No significant differences as a function of sex were found between experimental and control participants, χ^2^ = 0.26, *p* > 0.05. Twenty-five percent were 13 years old, 48.9% were 14, and 26.1% were 15.

A random sampling technique was used, applied to the list of schools in Gipuzkoa (Basque Country, Spain) and the type of school (public–private). Block randomization was performed by a computer-generated random-number list of schools prepared by the Department of Education of the Basque Government. The sample was recruited from three schools. Of these students, 44.3% attended public schools, and 55.7% private centers. The sampling unit was the school class.

The most recent survey of the Basque Statistical Institute was consulted to obtain a representative sample of Secondary Education students, confirming a population of 25,039. With a 0.90% confidence level and a sample error of 0.05, the representative sample comprises 173 adolescents. A prior power analysis was performed to determine sample size, presuming a low-medium effect size (*f* = 0.25), with a power of 0.90 (α = 0.05; 1 – β = 0.90) for the univariate *F* tests among the dependent variables, finding a minimum sample size of 171 participants (Faul et al., 2009).

The Basque Country, or Euskadi, is an Autonomous Community located in northern Spain (extension: 7,234 km^2^, population: 2,164,311 inhabitants). It exceeds the average European expenditure for R&D&I/GDP in innovation and its human development index of 0.96, as calculated by the Basque Institute of Statistics with methodology from the UN, represents a very high level. With regard to the availability of ICT in Basque schools, the report of the Department of Education of the Basque Government (Gobierno Vasco, 2014–2015) confirms that 100% of schools have Internet access; the percentage of ordinary classrooms with Internet access, of computerized ordinary classrooms (equipped with digital whiteboards, computers for teachers and students, interconnected and with Internet access) is 94.19, and 97% of the schools have WIFI access to the local network.

### Procedure

The study used a repeated measures pre-posttest design with a control group. The independent variable was the intervention program and the dependent variables were “school violence” and “premeditated and impulsive aggressiveness.” The procedure was phased as follows: (1) A letter was sent to the directors of the randomly selected schools from the list of schools in Gipuzkoa, explaining the project and requesting their collaboration; (2) Interviews were held with those directors who agreed to collaborate to present the project and distribute the informed consent forms for parents of the study participants; if the director of the selected center refused to collaborate, the procedure was repeated with the next school on the list, taking into account the type (public–private) and/or the socio-economic-cultural level of the school that declined to participate; (3) After receiving the parents’ consent, we administered the pretest to both experimental and control participants, using two assessment instruments to measure the dependent variables that the program was expected to affect; (4) Subsequently, the intervention program was applied in the five experimental groups (19 one-hour sessions), while the four control groups received the regular tutorship program of their school; and (5) After the intervention, at the posttest phase, we administered the same instruments as at pretest to both experimental and control groups.

The study complied with the ethical values required for research with humans (informed consent and the right to information, protection of personal data and guarantees of confidentiality, non-discrimination, gratuity, and the possibility to withdraw from the study at any phase), and received the favorable report of the University Research and Teaching Ethics Committee of the University of the Basque Country (CEISH/112/2012).

### Cyberprogram 2.0: An Intervention Program to Prevent and Reduce Cyberbullying

The program comprises activities aimed at preventing and/or intervening in bullying situations. The intervention consisted of 19 one-hour sessions carried out during the school term. The activities that make up the program have four main goals: (1) to identify and conceptualize bullying/cyberbullying, and the three roles involved in this phenomenon; (2) to analyze the consequences of bullying/cyberbullying for victims, aggressors, and observers, promoting critical capacity and the capacity for reporting these actions when they are discovered; (3) to develop coping strategies to prevent and reduce bullying/cyberbullying behaviors; and (4) other transversal goals, such as developing positive variables (empathy, active listening, social skills, strategies to control anger-impulsivity, constructive conflict resolution, tolerance in accepting a diversity of opinions, etc.).

The program was designed for administration to groups of adolescents by a teacher, psychologist, or school pedagogue. The program’s 25 activities are distributed in three intervention modules or axes about bullying and cyberbullying (see **Table 1**).

**Table 1:** Cyberprogram 2.0 modules and activities.

Modules	Activities
Module 1. Conceptualization and identification of roles	The cyberbullying corner
	Guess the Word 2.0.
	Collage
	Who’s who?
	Colored post-its
Module 2. Consequences, rights and responsibilities	Secrets from cyber-rooftops
	Sexting and false promises
	Posters
	Social networks
	Don’t trust completely
Module 3. Coping strategies	Jokes aside
	Megan Meier and Ryan Halligan
	Let’s talk about Patty
	Problem-solving: What can victims do?
	Break the law of silence
	Responding to aggressors
	Signing a contract
	Block Internet bullying
	Inspector Gadget
	I see, I see; what do you see?
	The impact of cyberbullying
	Photo comic
	Creating a blog
	Film-forum
	Visit to the Museum
	Cooperative Cyber-educate 2.0


#### Module 1: Conceptualization and Identification of Roles

This module is made up of five activities to help the group identify and discriminate the differences between bullying and cyberbullying in a specific situation. The aim of this module is for the group to acquire the necessary knowledge to be able to: (1) identify and define different types of bullying and cyberbullying, (2) analyze the differences between the two phenomena, and (3) know the main roles involved in these types of behaviors.

#### Module 2: Consequences, Rights, and Responsibilities

This module is made up of five activities aimed at analyzing the direct and indirect consequences of bullying and cyberbullying. It is important to understand what is happening, what the victims are feeling, what effects it has on all the people involved…, to finally develop competencies that inhibit such behaviors.

#### Module 3: Coping Strategies

This module contains 15 activities whose aim is to analyze the performance patterns of bullying situations from the viewpoint of all three roles involved (victim, aggressor, observer) to prevent, cope with, solve, eliminate, or minimize the effects of this type of violence. In other words, it consists of applying the knowledge acquired to seek plausible and effective solutions to situations of bullying and cyberbullying.

It is important to observe that one of the key aspects of the intervention program to prevent and cope with bullying and cyberbullying is training the participants to confront and know the consequences of this type of bullying. Accordingly, it is increasingly important to develop a series of transversal competences that implicitly complement each module in the enhancement of group processes and the generation of personal and social skills to inhibit bullying and cyberbullying. These goals are transversal to all three intervention modules. Summing up, the intervention program advocates for the development of a critical capacity, providing participants with the values of active listening and respect for others, which in turn lead to common good and democratic coexistence.

Each activity is described in a technical sheet that summarizes its specifications to simplify its implementation. The technical sheets of the program activities describe various parameters: (1) Goals: the specific goals of the activity; (2) Activity: the guidelines that the adult should offer the group for its development, and the implementation phases and procedure to be followed by the group; (3) Discussion: proposed questions or suggestions to promote debate or discussion; (4) Materials: resources needed to carry out the activity are described. A download link is provided when the activities involve viewing a video on YouTube, and the CD attached to the manual includes a file with all of the links to videos used when implementing Cyberprogram 2.0, in addition to dozens of links to videos about bullying, cyberbullying, social networks, sexting, grooming, Internet safety…. for use if the adult wishes to temporarily expand the implementation of this intervention; (5) Approximate duration of the activities; and (6) Group structure or how to organize the group in each activity (individuals, pairs, teams, large group). The manual includes the technical cards of the activities, the program’s implementation methodology and the assessment instruments.

The program’s group application entails the four constant variables of the intervention’s methodological framework: (1) *inter-session constancy*, which implies performing a weekly 1-h session; (2) *spatial-temporal constancy*, as the program is applied on the same weekday, at the same time, and in the same physical space (a large room free of obstacles such as a gymnasium, etc.); (3) *constancy of the adult* who directs the program, who must have psychopedagogical training; and (4) *constancy of the session structure*. The sessions begin with the group members sitting in a circle on the floor. The adult explains the activity, its goals, etc., and the participants carry out the action. Subsequently, there is a discussion and guided reflection phase, led by the adult. The adult promotes critical reflection through the formulation of non-judgmental questions. All sessions follow this structure, except for the first one in which the intervention program, its goals, duration, and types of activities to be implemented are presented and explained. The program uses diverse group dynamics techniques to stimulate the development of the activity and debate: role-playing, brainstorming, case study, guided discussion by means of formulating questions, and so on.

For example, Activity 14. *Should aggressors be punished?* Firstly, we showed the video “X Nada” [For no reason] (http://www.youtube.com/watch?v=REA80mMaCsY), which tells the story of a group of adolescents who beat up another teenager, who must be hospitalized as a result. The attack is filmed with a mobile phone and uploaded to the Internet. In this video, an adolescent suffers an attack of “happy slapping” carried out by Roy, the aggressor, and his gang of pals. After seeing the video (14 min), teams of five people are formed and they debate about whether the aggressors should be punished and what should be done to eradicate their aggressive behavior. For this purpose, each team names a secretary who records all the members’ suggestions to solve the problem. Subsequently, by turns, each team selects by consensus the most efficacious and constructive way to penalize the aggressor of bullying/cyberbullying and dramatizes the scene by representing the chosen solution.

After the representations, sitting on the floor in a circle, the entire group discusses the responses of each team, and a debate is initiated in which the pros and cons of the teams’ proposed responses are analyzed, clarifying which are more efficient and positive to inhibit aggressive behaviors in perpetrators of bullying and cyberbullying. The published program manual (Garaigordobil and Martínez-Valderrey, 2014a) presents the activities and methodology for implementation with a group, therefore enabling the study’s replication.

### Assessment Instruments

We administered two assessment instruments with psychometric guarantees of reliability and validity to assess the effects of the intervention before and after the program.

“*Cuestionario de Violencia Escolar Revisado*” (*CUVE-R* [Revised Questionnaire of School Violence]; Álvarez-García et al., 2011). This instrument explores adolescents’ perception of diverse types of school violence: teachers’ violence toward students, students’ physical and verbal violence, social exclusion, disruption in the classroom, violence by means of ICT. It comprises 31 items with a 5-point Likert-type response scale, ranging from 1 (*it never occurs)* to 5 (*it always happens to me).* For example: “Some students film or take pictures of classmates with their mobile to make fun of them.” “The teachers punish unfairly.” “Some students hit other classmates when they’re on the school grounds.” “Some students badmouth other students.” The Cronbach alpha coefficient obtained with the original standardization test sample confirmed high internal consistency (overall scale: α = 0.92; factors: ranging from α = 0.87 for teachers’ violence toward students to α = 0.67 for direct physical violence among students). The coefficients obtained with the study sample were higher (overall scale: α = 0.94; teachers’ violence toward students: α = 0.88; students’ physical violence: α = 0.80; students’ verbal violence: α = 0.80; social exclusion: α = 0.63; disruption in the classroom: α = 0.80; violence through ICT: α = 0.83). The exploratory and confirmatory analyses revealed a 6-factor model. The standardized regression coefficient showed statistically significant factor loadings.

“*Cuestionario de agresividad premeditada e impulsiva en adolescentes*” (*CAPI-A* [Adolescents’ Premeditated and Impulsive Aggressiveness Questionnaire]; Andreu, 2010). Aggressiveness is expressed in different forms: physical or psychological, active or passive, direct or indirect, and so on. The CAPI-A assesses premeditated aggressiveness (an instrumental means aimed at obtaining a goal other than harming the victim) and impulsive aggressiveness (referring to an unplanned response, derived from anger as a result of perceived provocation and with the intention of harming the victim). The questionnaire is made up of 24 statements on which respondents rate their degree of agreement on a 5-point Likert scale ranging from 1 (*strongly disagree*) to 5 (*strongly agree*). For example, “I think my aggressiveness is justified.” “When I get mad, I react without thinking.” “Being aggressive has helped me gain power over others and improved my social level.” Psychometric studies confirm the reliability of the Premeditated and Impulsive Aggressiveness scales, α = 0.83 and α = 0.82, respectively. The coefficients obtained with the study sample were similar (α = 0.82). The CAPI-A correlated positively with impulsivity and with reactive/proactive aggressiveness, thereby ratifying its convergent validity. Premeditated aggressiveness presented a slightly higher correlation with proactive aggressiveness, and impulsive aggressiveness had a higher correlation with reactive aggressiveness.

### Data Analysis

To assess the program’s effect on the dependent variables, firstly, we carried out descriptive analyses (means and standard deviations) and univariate and multivariate analyses of variance (ANOVA and MANOVA) with the pretest scores obtained on the CUVE-R (school violence) and the CAPI-A (aggressiveness) by the experimental and control participants. Secondly, we carried out descriptive analyses and analyses of covariance of the pre-posttest differences (pre-posttest MANCOVA, ANCOVA) using the pretest differences between the two conditions as covariate, thereby determining the intervention’s impact. In addition, to analyze whether the change was similar in males and females, first, we performed ANOVAs with the pretest scores and, subsequently, ANCOVAs of the pre-posttest differences in the dependent variables of both sexes.

## Results

### Effects of the Program on School Violence and Aggressiveness

With regard to school violence, firstly, we performed a MANOVA on the pretest CUVE-R scores of the experimental and control groups. The results of this pretest MANOVA on the variables school violence (teachers’ violence toward students, students’ physical and verbal violence, social exclusion, disruption in the classroom, violence through ICT) showed statistically significant group differences before the intervention, Wilks’ lambda, Λ = 0.798, *F*(6,169) = 7.12, *p* < 0.001, with a small effect size (η^2^ = 0.202, *r* = 0.44). The descriptive analyses of each variable and the results of the pretest ANOVA (see **Table 2**) revealed statistically significant group differences in all types of school violence before implementing the program, with the experimental participants scoring higher than the control group. Except for the variables social exclusion and disruption in the classroom, which had small effect sizes, the effect sizes of the remaining variables were moderate and high.

**Table 2:** Means, standard deviations, results of the pretest ANOVAs, of pre-posttest ANCOVAs, and effect size (d) in school violence and aggressiveness in experimental and control groups.

	Pretest				Pre-posttest Differences				Pretest ANOVA			Pre-posttest ANCOVA		
			
	Experimental		Control		Experimental		Control							
	
	*M*	*SD*	*M*	*SD*	*M*	*SD*	*M*	*SD*	*F*(1,174)	*p*	*d*	*F*(1,174)	*p*	*d*
Teachers’ violence toward students	16.56	6.48	12.67	4.79	–1.96	5.70	1.91	4.79	20.00	0.000	0.68	7.43	0.007	-0.73
Students’ physical violence	13.22	4.56	10.55	3.77	–1.59	4.33	1.12	4.08	17.48	0.000	0.63	4.34	0.039	-0.64
Students’ verbal violence	15.16	4.58	11.66	3.56	–2.75	4.71	1.46	3.93	31.43	0.000	0.85	11.49	0.001	-0.97
Social exclusion	5.40	1.88	4.81	1.79	–0.98	1.86	0.49	1.90	4.50	0.035	0.32	21.55	0.000	-0.78
Disruption in the classroom	8.26	2.94	7.11	2.71	–0.35	3.14	0.54	2.77	7.18	0.008	0.40	0.09	0.763	-0.30
ICT violence	9.63	4.08	7.45	2.18	–2.41	3.04	1.00	3.46	18.97	0.000	0.66	33.56	0.000	-1.04
Total violence	68.23	19.00	54.25	15.57	–6.31	12.51	4.57	10.59	28.03	0.000	0.80	11.66	0.001	-0.93

Premeditated aggressiveness	29.05	7.56	25.45	6.43	-6.09	10.38	1.38	8.66	11.34	0.001	0.51	31.14	0.000	-0.78
Impulsive aggressiveness	32.38	10.25	30.18	8.64	-9.21	10.46	-1.76	8.02	2.30	0.131	0.22	34.85	0.000	-0.79


Secondly, to assess the program’s efficacy in diverse types of violence, we analyzed the pre-posttest change. The results of the MANCOVA of the pre-posttest difference of means for all the variables of school violence revealed statistically significant group differences in change, Wilks’ lambda, Λ = 0.664, *F*(7,167) = 12.09 *p* < 0.001), with a medium effect size (η^2^ = 0.336, *r* = 0.57). With regard to each type of school violence, we conducted descriptive analyses and ANCOVA of the pre-posttest differences in both conditions, with the pretest scores as covariate. The results of the ANCOVAs (see **Table 2**) confirmed statistically significant group differences in pre-posttest change in all the variables of school violence measured, except for disruption in the classroom. The effect size was large for all variables. Examination of the change produced in the experimental and control participants (see **Table 2**) showed that, whereas the experimental group mean (*Me*) decreased significantly in almost all the variables of school violence, the control group mean (*Mc*) increased: teachers’ violence toward students (*Me* = –1.96, *Mc* = 1.91), students’ physical violence (*Me* = –1.59, *Mc* = 1.12), students’ verbal violence (*Me* = –2.75, *Mc* = 1.46), social exclusion (*Me* = –0.98, *Mc* = 0.49), violence through ICTs (*Me* = –2.41, *Mc* = 1.00), and total violence (*Me* = –6.31, *Mc* = 4.57).

Regarding aggressiveness, firstly, we conducted a MANOVA of the pretest scores of the CAPI-A. The MANOVA results for aggressiveness, Wilks’ lambda, Λ = 0.936, *F*(2,172) = 5.90, *p* < 0.01, showed statistically significant group differences before the intervention, with a small effect size (η^2^ = 0.064, *r* = 0.25). The descriptive analyses of each variable and the results of the pretest ANOVA comparing the two conditions (see **Table 2**) indicated statistically significant group differences only in premeditated aggressiveness, with higher scores in the experimental group, and a moderate effect size.

Secondly, to assess the program’s impact on aggressiveness, we analyzed the pre-posttest change. The results of the MANCOVA of the pre-posttest difference of means in aggressiveness showed significant group differences in change, Wilks’ lambda, Λ = 0.859, *F*(2,171) = 13.99, *p* < 0.001, with a small effect size (η^2^ = 0.141, *r* = 0.37). Complementarily, as regards each type of aggressiveness (impulsive and premeditated), we conducted descriptive analyses and ANCOVA of the pre-posttest group differences, using the pretest scores as covariate. The results of the ANCOVA (see **Table 2**) confirmed statistically significant group differences in pre-posttest change both in premeditated and impulsive aggressiveness. The effect size was large. Comparison of the change produced in the two conditions (see **Table 2**) revealed that the experimental group decreased its mean (*Me*) significantly more than the control group (*Mc*) in premeditated (*Me* = –6.09, *Mc* = 1.38) and impulsive aggressiveness (*Me* = –9.21, *Mc* = –1.76).

### Effects of the Program on School Violence and Aggressiveness in Both Sexes

The descriptive analyses of each variable and the results of the pretest and pre-posttest ANOVAs by sex (see **Table 3**) revealed no statistically significant differences between males and females in all the variables of school violence assessed. Therefore, there were no sex differences before the intervention, and the change due to the effect of the program was similar in both sexes. Regarding aggressiveness, the results of the pretest and pre-posttest ANCOVAs by sex (see **Table 3**) also showed that there were no sex differences before the intervention, and that the pre-posttest change was similar in premeditated aggressiveness; however, females decreased impulsive aggressiveness significantly more than males.

**Table 3:** Means, standard deviations, results of pretest ANOVAs, of pre-posttest ANCOVAs and effect size (d) of different types of school violence, in males and females.

	Pretest				Pre-posttest Differences				Pretest ANOVA			Pre-posttest ANCOVA		
			
	Males		Females		Males		Females							
	
	*M*	*SD*	*M*	*SD*	*M*	*SD*	*M*	*SD*	*F*(1,91)	*p*	*d*	*F*(1,91)	*p*	*d*
Teachers’ violence toward students	16.95	7.02	16.28	6.12	–2.36	6.55	-1.58	5.02	0.24	0.625	0.10	0.08	0.769	0.13
Students’ physical violence	12.95	5.19	13.41	4.09	–1.92	5.04	-1.32	3.75	0.22	0.635	0.09	2.28	0.135	0.13
Students’ verbal violence	14.44	5.10	15.69	4.12	–2.92	5.31	-2.62	4.25	1.69	0.196	0.26	2.36	0.128	0.06
Social exclusion	5.08	2.01	5.63	1.76	–0.82	1.98	-1.09	1.79	1.97	0.164	0.29	0.05	0.818	0.14
Disruption in the classroom	8.13	3.74	8.35	2.23	–0.15	3.24	-0.51	3.08	0.12	0.720	0.07	0.07	0.783	0.11
ICT violence	8.87	4.23	10.19	3.91	–1.18	3.58	-0.96	2.60	2.37	0.127	0.32	0.01	0.908	0.07
Total violence	66.41	21.92	69.54	16.68	–6.36	14.05	-6.21	11.39	0.61	0.437	0.16	0.39	0.534	0.01

Premeditated aggressiveness	29.10	7.65	29.02	7.57	–7.62	9.54	-11.04	10.81	0.00	0.958	0.01	2.28	0.134	0.11
Impulsive aggressiveness	31.59	9.12	32.94	11.04	–6.44	10.05	-11.53	10.33	0.39	0.532	0.13	6.44	0.013	0.49


## Discussion

The aim of the study was to assess the effects of an antibullying program on school violence and aggressiveness. Firstly, the results obtained confirm that the program had a very positive effect, as it promoted a decrease of school violence: (a) *teachers’ violence toward students* (the teacher ridicules, publicly humiliates a student in front of the class, makes fun of, despises, or treats one or various students differently from the others); (b) *students’ physical violence* (fights, blows, shoves… aimed at the victim, or at his or her property, such as theft, hiding possessions); (c) *students’ verbal violence* (using offensive language, cruel, embarrassing, or insulting words, aggressive tone of voice… toward classmates and teachers); (d) *social exclusion* (acts of discrimination, rejection, or exclusion aimed at a person or group, because of physical aspect, academic performance, sexual condition, socioeconomic-cultural status, sex, race, etc.); and (e) *violence through ICT or cyberbullying* (violent behaviors by means of electronic instruments such as mobile phones and the Internet). Therefore, the results allow us to confirm Hypothesis 1 almost completely because the program decreased almost all types of school violence; no differences were found only in behaviors of disruption in the classroom. Secondly, the results show that the experimental group significantly decreased premeditated aggressiveness and impulsive aggressiveness. Hence, the data also confirm Hypothesis 2.

Summing up, the results show that the program significantly decreased behaviors related to the different types of school violence: physical, verbal, social, and technological violence among students and by teachers toward students. In addition, it stimulated a decrease in impulsive and premeditated aggressiveness. These results point in the same direction as other studies showing the efficacy of antibullying interventions to improve the social climate of the classroom and the school (Olweus, 1991; Olweus and Limber, 2010a,b; Rawana et al., 2011), intragroup relations (Fekkes et al., 2006), and the feeling of safety at school (Heydenberk et al., 2006), and to decrease aggressiveness (Grossman et al., 1997; McMahon et al., 2000; Ortega and Lera, 2000; Orpinas et al., 2003; Fonagy et al., 2005; Twemlow et al., 2005). Moreover, the results also confirm the positive effects of other programs for the prevention of cyberbullying (Doane, 2011; Lee et al., 2013; Chaux et al., 2016).

Thirdly, the results have revealed that the change stimulated by the intervention program was similar in males and females for all the variables of school violence assessed and for premeditated aggressiveness. However, in impulsive aggressiveness, females decreased significantly more than males. Therefore, Hypothesis 3 is confirmed almost completely, pointing in the same direction as other studies showing that intervention programs to promote socio-emotional development and prevent violence affect both sexes similarly (Garaigordobil et al., 2009; Garaigordobil and Peña-Sarrionaindia, 2015).

The program’s activities create and structure situations of communication, cooperation, and empathy that explain the intervention’s positive effects in decreasing violent behaviors (diverse types of school violence and aggressiveness). This decrease may be explained by the emphasis of the Cyberprogram 2.0 on activities concerning the feelings of those involved, and particularly, the emotional experience of the victim. Many program activities emphasize the negative consequences of bullying for victims, perpetrators, and observers, the responsibility of observers, and critical analysis of aggressors. This is consistent with a previous evaluation (Garaigordobil and Martínez-Valderrey, 2014b) that confirmed that Cyberprogram 2.0 stimulated a significant decrease in victimization and an increase of positive social behaviors (social conformity, help-collaboration, self-assurance-firmness, prosocial leadership).

The results show the therapeutic effect of this type of intervention. This role of the school as a context for development has already been pointed out by other researchers (Mestre et al., 2012). The prosocial behavior stimulated by the activities, the debates dealing with various relevant themes (such as the negative effect of aggressive behavior on others), or the reinforcement received from some group members for their positive social behaviors (helping each other, listening to one another, cooperating, etc.)—all of this contextualized within the cognitive-behavioral theoretical framework—may explain the positive effects derived of the program.

From the viewpoint of psychological development, previous assessment studies of Cyberprogram have confirmed that the program’s experimental participants increased their capacity for communication, empathy, skills for assuming another’s perspective, confronting and resolving interpersonal conflicts constructively, self-esteem… (Garaigordobil and Martínez-Valderrey, 2014b, 2015a,b), which led to greater personal maturity, resulting in improved social behaviors. Over the course of the program, cognitive restructuring (elimination of erroneous and distorted beliefs about victims and aggressors) is stimulated, enhancing behavioral changes that inhibit violent behavior (for example, constructive coping with the bullying situation by the victim and the observers, as well as control of the aggressors’ negative behavior, etc.).

Among the possible reasons for greater program efficiency are: (1) the time allocated to the intervention (changing negative behaviors requires time, and some programs offer few sessions); (2) the characteristics of the included activities (more effective programs include activities that stimulate empathy, analysis of the consequences of violence for all involved, peaceful conflict resolution, group members’ self-esteem, anger control…); (3) the involvement and training of the teachers who implement the program (the higher their degree of involvement and training, the more efficient the program); and (4) the parents’ participation (their involvement reinforces the effects of the psychoeducational intervention). Therefore, educational authorities should raise awareness among the entire school community (teachers, parents, non-teaching staff…) through awareness-raising campaigns, training teachers to implement programs that inhibit violent behavior, and seeking financial support for the implementation of these proposals.

As reported when describing the study’s participants and context, the students of the Basque Country have a high level of access to ICT, and previous studies (e.g., Tokunaga, 2010) have shown that access to ICT is related to a higher probability of being a cybervictim and/or a cyberaggressor. In a recent epidemiological study carried out in the Basque Country with students aged 12–18 years (Garaigordobil, 2015), it was highlighted that, in the past year, 30.2% had experienced cyberbullying (cybervictims) one or more times, 15.5% had perpetrated one or more cyberbullying behaviors against others (cyberaggressors), and 65.1% had observed classmates carrying out cyberbullying behaviors against others (observers). Thus, given its noteworthy prevalence in the Basque Country, the intervention to reduce it is shown to be necessary and may partially explain the positive result of Cyberprogram 2.0.

## Conclusion

The conclusions drawn from this study have interesting implications for educational and clinical intervention with all adolescents involved in bullying/cyberbullying situations, especially with aggressors. This work provides empirical evidence showing that an antibullying program can have a very positive impact on different types of school violence and different kinds of aggressive behavior.

Altogether, the results allow us to emphasize the importance of implementing programs during childhood and adolescence to promote socio-emotional development, improve coexistence, and prevent/reduce violence. The best way to prevent violence is through the promotion of harmonious coexistence, and Cyberprogram 2.0, an evidence-based intervention program to prevent cyberbullying, is proposed within this framework. All schools should define an action protocol for cases of harassment and a plan to prevent violence and promote peaceful coexistence. All students should participate in preventive programs to inhibit all modes of violence. In addition, didactic proposals must be implemented for the family for both prevention and intervention in violence.

The use of self-reports, with the inherent bias of social desirability, is a limitation of the study. For future research, we suggest using hetero-reports in which parents and teachers inform of adolescents’ attitudes and behaviors and/or observational techniques to assess and ratify the program’s effects. In addition, the research sample size is insufficient to provide results generalizable to the population. Therefore, we suggest an assessment of the program with a larger sample size and of broader origin. Future lines of research could analyze the connections of cyberbullying with personal (personality traits, psychopathological symptoms, behavioral problems, etc.) and family factors (parental socialization styles), that could help to identify relevant variables for prevention and intervention programs. In addition, an interesting future challenge would be to analyze the effect of the characteristics and training of the adult who directs the intervention on the changes stimulated by Cyberprogram in the adolescent participants.

## Author Contributions

MG has designed research and the program. Also she has carried out the data analysis and has written the article. VM-V has designed the program and has implemented the program with adolescents.

## Conflict of Interest Statement

The authors declare that the research was conducted in the absence of any commercial or financial relationships that could be construed as a potential conflict of interest.
